# Resident Fibroblast MKL1 Is Sufficient to Drive Pro-fibrogenic Response in Mice

**DOI:** 10.3389/fcell.2021.812748

**Published:** 2022-02-01

**Authors:** Shan Huang, Tinghui Shao, Hong Liu, Tianfa Li, Xianhua Gui, Qianwen Zhao

**Affiliations:** ^1^ Key Laboratory of Targeted Intervention of Cardiovascular Disease and Collaborative Innovation Center for Cardiovascular Translational Medicine, Department of Pathophysiology, Nanjing Medical University, Nanjing, China; ^2^ Hainan Provincial Key Laboratory for Tropical Cardiovascular Diseases Research, Key Laboratory of Emergency and Trauma of Ministry of Education, Department of Cardiology, Research Unit of Island Emergency Medicine of Chinese Academy of Medical Sciences, The First Affiliated Hospital of Hainan Medical University, Haikou, China; ^3^ Department of Respiratory Medicine, Affiliated Nanjing Drum Tower Hospital, Nanjing University School of Medicine, Nanjing, China

**Keywords:** fibrosis, fibroblast, transcription factor, wound healing, MKL1 (MRTF-A)

## Abstract

Fibrosis is an evolutionarily conserved pathophysiological process serving bifurcated purposes. On the one hand, fibrosis is essential for wound healing and contributes to the preservation of organ function. On the other hand, aberrant fibrogenic response may lead to tissue remodeling and precipitate organ failure. Recently lineage tracing studies have shown that resident fibroblasts are the primary mediator of fibrosis taking place in key organs such as the heart, the lungs, and the kidneys. Megakaryocytic leukemia 1 (MKL1) is transcriptional regulator involved in tissue fibrosis. Here we generated resident fibroblast conditional MKL1 knockout (CKO) mice by crossing the *Mkl1* ^f/f^ mice to the *Col1a2*-Cre^ERT2^ mice. Models of cardiac fibrosis, pulmonary fibrosis, and renal fibrosis were reproduced in the CKO mice and wild type (WT) littermates. Compared to the WT mice, the CKO mice displayed across-the-board attenuation of fibrosis in different models. Our data cement the pivotal role MKL1 plays in tissue fibrosis but point to the cellular origin from which MKL1 exerts its pro-fibrogenic effects.

## Introduction

Fibrosis is considered a key part of the wound healing process that safeguards the architectural and functional integrity of the host (Henderson et al., 2020). On the one hand, fibrotic tissue (scar) helps cover the wound to prevent excessive loss of blood and maintain body fluid homeostasis. On the other hand, accelerated production of extracellular matrix (ECM) proteins contributes to interstitial remodeling and preservation of organ function. In order to fulfill these requirements, a specialized cell type emerging from the injured tissues, termed “myofibroblast”, is able to both perform muscle-like contraction and synthesize ECM proteins (Pakshir et al., 2020). Spatiotemporally controlled activation and deactivation of myofibroblasts, acting as the chief effector of fibrosis, ensures proper initiation and termination of the fibrogenic response. Dysregulation of myofibroblast maturation and resolution, on the contrary, is invariably associated with the loss of key organ function. For instance, insufficient activation of myofibroblasts in the aging heart triggers defective wound healing and heart failure following myocardial infarction (Bujak et al., 2008). Alternatively, depletion of excessive myofibroblasts mitigates adverse interstitial remodeling and improves organ function in mice (Kaur et al., 2016; Meng et al., 2016; Xu et al., 2020).

Megakaryocytic leukemia 1 (MKL1) is a transcriptional regulator initially identified and characterized by Eric Olson and colleagues as a co-factor for serum response factor (SRF) to regulate the transcription of contractile genes (Wang et al., 2002). Early studies have found that over-expression of MKL1 is sufficient to stimulate the transcription of contractile genes in non-muscle cells indicating that MKL1 is the rate-limiting factor, at least under certain circumstances, in the acquisition of a contractile phenotype (Zhang et al., 2007). On the contrary, global MKL1 knockout (KO) mice are indistinguishable from their wild type (WT) littermates in terms of smooth muscle contraction or contractile gene expression in physiological settings (Li et al., 2006; Sun et al., 2006), which again seems to allude to the notion that MKL1 is required for the acquisition, rather than the maintenance, of a contractile phenotype. Indeed, research in the past decade has provided mounting evidence that MKL1 is a master regulator in tissue fibrosis. Small et al. have presented the first *in vivo* evidence that the global MKL1 KO mice display significantly weakened cardiac fibrosis compared to the WT mice in a model of myocardial infarction (Small et al., 2010). This report was followed by a string of studies that implicate MKL1 in the regulation of pulmonary fibrosis (Zhou et al., 2013), scleroderma (Shiwen et al., 2015), liver fibrosis (Fan et al., 2015; Tian et al., 2015; Wu X. et al., 2020), renal fibrosis (Xu et al., 2015; Mao et al., 2020b), and intestinal fibrosis (Johnson et al., 2014). Despite these observations, it is not clear from which cell type MKL1 exerts its pro-fibrogenic effects due to its universal expression pattern (Olson and Nordheim, 2010). For instance, Weng et al. have previously that mice with endothelial cell-specific MKL1 deletion are partially resistant to angiotensin II (Ang II) induced cardiac fibrosis and heart failure (Weng et al., 2015). Similarly, Liu et al. have recently reported that conditional deletion of MKL1 from the myeloid lineage protects the mice from pressure overload induced pathological hypertrophy and cardiac fibrosis in mice (Liu et al., 2021a).

Recent advances in genetic lineage tracing technique have greatly facilitated the attempt to uncover the origin(s) of myofibroblasts in tissue fibrosis. Although substantial variations exist in the accumulated data depending on both the type of tissue examined and the method used to induce fibrosis, there appears to be a consensus that resident fibroblasts represent a predominant source for myofibroblast in the heart (Kanisicak et al., 2016), in the kidneys (LeBleu et al., 2013), and in the lungs (Zepp et al., 2017). Based on these results, we hypothesized that MKL1 deficiency in tissue resident fibroblasts may be sufficient to influence fibrosis. Our data demonstrate that conditional MKL1 deletion in resident fibroblasts attenuates myocardial fibrosis, renal fibrosis, and pulmonary fibrosis *in vivo*.

## Methods

### Animals

All animal protocols were reviewed and approved the intramural Ethics Committee on Humane Treatment of Laboratory Animals of Nanjing Medical University. The mice were maintained in an SPF environment with 12 h light/dark cycles and libitum access to food and water. Resident fibroblast specific MKL1 knockout mice were created by crossing by the *Mkl1* ^f/f^ mice (Liu et al., 2021a; Liu et al., 2021b) with the *Col1a2*-Cre^ERT2^ mice (Wang et al., 2016). Cardiac fibrosis was induced by permanent ligation of left anterior descending coronary artery as previously described (Yang et al., 2017). Renal fibrosis was induced by the unilateral ureteral obstruction procedure as previously described (Mao et al., 2020a; Mao et al., 2020b). Pulmonary fibrosis was induced by intratracheal instillation of bleomycin as previously described (Xu et al., 2011; Ianni et al., 2021).

### Bronchoalveolar Lavage Fluid

7 days following intratracheal bleomycin administration or 0.9% saline treatment, BALF was performed. The mice were sacrificed in deep anesthesia by atlanto-occipital dislocation. The trachea was exposed and a catheter was used to intubate the mice. The lungs were instilled 3 times using 1 ml of 0.9% saline. The BALF was collected, centrifuged at 400 g for 10 min, the total cells in the pellet were collected for detecting RNA levels, while BALF was preserved at −20°C.

### Urine Analyses

Urine samples were collected from the mice after 14-days UUO surgery. Urinary creatinine (Cr) and urea nitrogen (BUN) were quantified separately using commercial assay kits from Nanjing Jiancheng Bioengineering Institute (Nanjing, China) according to the manufacturer’s instructions.

### Cardiac Function Assessment by Echocardiography

A non-invasive transthoracic echocardiographic examination was performed at the 21st day after ligation of left anterior descending coronary artery by using a Vevo 2,100 (Visual sonics, Canada), equipped with a 30-MHz transducer. The mice were anesthetized by 2% isoflurane gas (1.5% with O_2_ 1 L/min). Two-dimensional guide M-mode tracings were recorded, and the ejection fraction (EF), fractional shortening (FS), late diastolic transmitral flow velocity (E’/A′) and early diastolic mitral annular velocity (E/E’) were measured or further calculated.

### RNA Isolation and Real-Time Quantitative Polymerase Chain Reaction (RT-qPCR) Analysis

RNeasy RNA isolation kit (Qiagen) was used to extract total RNA from tissue sample of mice according to the manufacturer’s procedure. The extracted RNA was dissolved in diethypyrocarbonate (DEPC)-treated water, and the RNA concentration was determined by optical density measurement at 260 nm using a spectrophotometer. RNA sample (1 µg) was subsequently reverse transcribed using a HiScript III RT SuperMix (Vazyme). Real-time PCR reactions were carried out on an ABI Prism StepOne Plus system with a commercial SYBR green kit (Vazyme). The primers used were as follows: 18s: 5′- GGACCAGAG CGAAAGCATTTGCC-3’ (forward) and 5′-TCA​ATC​TCG​GGT​GGC​TGA ACGC-3’ (reverse); Acta2: 5′-CCT​GTT​TCG​GGA​GCA​GAA-3’ (forward) and 5′-GGT​TAT​ATA​GCC​CCC​TGG-3’ (reverse); Col1a1: 5′-GCT​CCT​CTT​AGG​GGC​CAC​T-3’ (forward) and 5′-CCACGTCTC ACCATTGGGG -3’ (reverse); Col3a1: 5′-TAG​TAA​CAT​GGA​AAC​CTG​GGG​AAA-3’ (forward) and 5′-CCA​TAG​CTG​AAC​TGA​AAA​CCA​CC-3’ (reverse). Ct values of target genes were normalized to the Ct values of the endogenous control 18s using the ΔΔCt method and expressed as relative mRNA expression levels compared to the control group which is arbitrarily set as 1.

### Western Blot Analysis

The total protein was extracted from the samples using radioimmunoprecipitation assay (RIPA) buffer (50 mM Tris pH7.4, 150 mM NaCl, 1% Triton X-100) with freshly added protease inhibitor tablet (Roche). Protein concentrations were quantified using a Bicinchoninic Acid Protein Assay kit (Biosky Biotechnology, Nanjing, China) according to the manufacturer’s protocol. Thirty micrograms of protein samples were separated by 8% sodium dodecyl sulfate-polyacrylamide gel electrophoresis (SDS-PAGE) and electrophoretically transferred to PVDF membranes (0.45 µm; EMD Millipore, Billerica, MA, United States). Membranes were blocked for 1 h at room temperature with 5% BSA (Sangon Biotech Co., Ltd.) in TBS-Tween-20 (containing 5 mM Tris-HCl (pH 7.6), 136 mM NaCl and 0.05% Tween-20). Then Membranes were incubated with anti-TBP (Proteintech, China, 22006-1-AP, 1:1,000), anti-MKL1 (Proteintech, China, 21166-1-AP, 1:1,000) overnight at 4°C. Subsequently, the primary antibodies were detected with secondary antibody (anti-Rabbit, Beyotime Institute of Biotechnology, China, A0208, 1:3,000) and scanned by using enhanced chemiluminescence western detection reagents (Thermo Fisher Scientific, Inc.). Image-Lab version 5.2.1 software (Bio-Rad Laboratories, Inc.) was used to quantify the intensity of the band in western blot assay.

### Masson, Sirius Red Staining and Hematoxylin/Eosin Staining

Histological analysis was performed with conventional methods as previously described (Li et al., 2018). Briefly, tissue samples were placed in 4% paraformaldehyde (PFA) in PBS overnight and then dehydrated in a gradient ethanol solution and embedded in paraffin. The tissue in the paraffin block was cut to a thickness of 5 μm.

HE staining was conducted according to routine protocols. Briefly, after deparaffinization and rehydration, the tissue sections were stained with hematoxylin solution for 5 min followed by immersed in 1% acid ethanol (1% HCl in 70% ethanol) for 15 s, and then rinsed in tap water for 10 min. Subsequently, the sections were stained with eosin solution for 2 min and followed by dehydration with graded alcohol and clearing in xylene. Pictures were taken using an Olympus IX-70 microscope (Olympus, Tokyo, Japan).

PicroSirius Red staining (Sigma-Aldrich) was conducted with PicroSirius Red kit. Sections were incubated in PicroSirius Red solution for 1 h, and then rinsed in distilled water. Subsequently, the dehydrated and mounted. Pictures were taken using an Olympus IX-70 microscope (Olympus, Tokyo, Japan) and polarizing microscope (Olympus VS200, Yokyo, Japan). The fibrotic area of each picture was analyzed by Image J software.

Masson’s trichrome staining was conducted with Trichrome Stain Kit (Sigma-Aldrich). Briefly, the paraffin-embedded sections were cut and placed on standard microscopy slides. After deparaffinization and rehydration, the slides were immersed in distilled water. Subsequently, the sections were stained in weigert’s hematoxylin for 5 min, and then washed again with tap water for 5 min and rinsed in distilled water. Next, the slides were stained in scarlet-acid fuchsin for 5 min, rinsed in 0.3% acetic acid, incubated in phosphomolybdic acid for 2 min, dyed with aniline blue for 5 min, and fixed in 0.3% acetic acid for 1 min. Finally, the slides were dehydrated and mounted. Pictures were taken using an Olympus IX-70 microscope (Olympus, Tokyo, Japan) and the fibrotic area of each picture was analyzed by Image J software.

### Immunofluorescence and Immunohistochemistry Staining

For immunofluorescence staining of MKL1, FSP1 and Col1a, the tissue slices were incubated with anti-MKL1 (Abcam, 1:200), anti-FSP1 antibodies (Proteintech, 1:100) and anti- Col1a (Abcam, 1:200) respectively, in one humidified chamber at 4°C overnight, followed by incubation with FITC-labeled secondary antibodies at 37°C for 1 h. Then, cell nuclei were stained with DAPI (Sigma) for 15 min. The immune stained images were captured using a confocal microscope (Olympus, Tokyo, Japan).

Immunohistochemistry was performed. Lung paraffin-embedded sections were permeabilized and blocked with 5% BSA, and then incubated with anti-CD68 (Proteintech, 1:100), and anti-CD45 (Proteintech, 1:100) respectively at 4°C overnight, then incubated with secondary antibody (Beyotime Institute of Biotechnology, 1:200) at room temperature for 1 h. Positive immunostaining was visualized by using the diaminobenzidine substrate (DAB, Thermo Fisher, United States) for 1 min. Next hematoxylin was utilized to stain nuclei for 20 s. The immune stained images were captured using a confocal microscope (Olympus, Tokyo, Japan).

### Statistical Analysis

All data were presented as means ± SD. Two group comparisons were made using two-tailed Student’s t tests. Multiple group comparisons were made using one-way analysis of variance. A significant difference between groups was considered as *p* values <0.05.

## Results

### Validation of Megakaryocytic Leukemia 1 Deletion in Resident Fibroblasts

**FIGURE 1:**
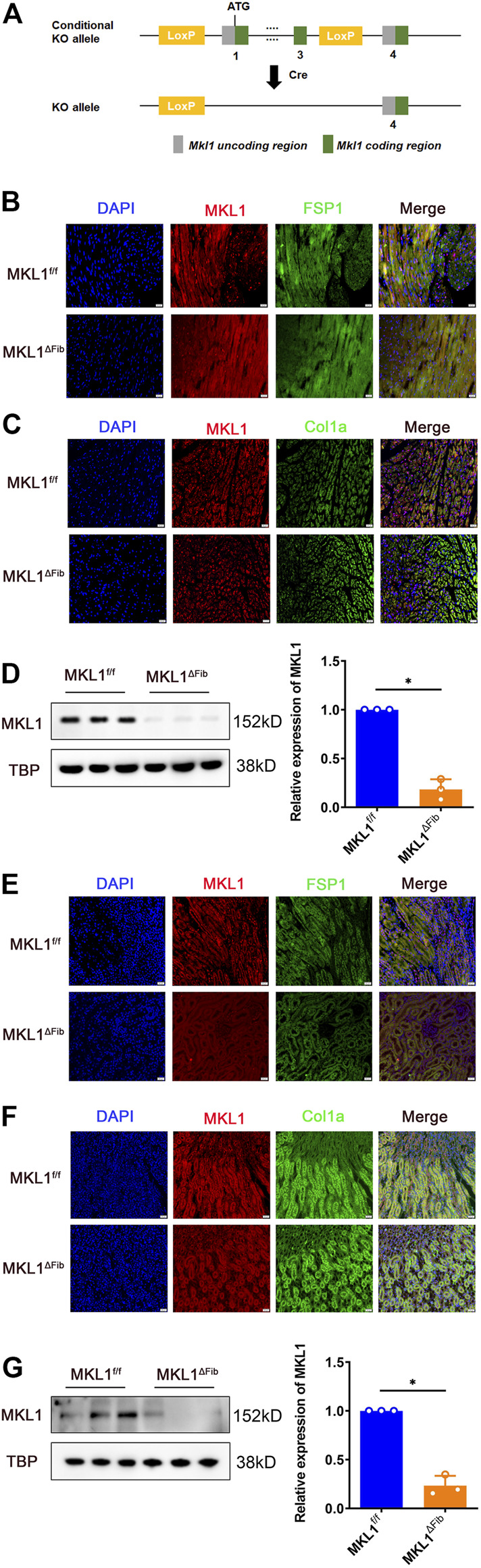
Validation of MKL1 deletion in resident fibroblasts. WT and CKO mice were injected with tamoxifen (50 mg/kg) for 10d. **(A)** Scheme of protocol. **(B)** MKL1^+^FSP1^+^ cells in cardiac tissue were measured by immunofluorescence staining. Scale bar, 20 μm. **(C)** MKL1^+^Col1a^+^ cells in cardiac tissue were measured by immunofluorescence staining. Scale bar, 20 μm. **(D)** primary cardiac fibroblasts were isolated from the MKL1^ΔFib^ heart and the MKL1^f/f^ heart, western blotting detected the MKL1 protein level. **(E)** Immunofluorescence staining showed the MKL1^+^FSP1^+^ cells in renal tissue. Scale bar, 20 μm. **(F)** Immunofluorescence staining showed the MKL1^+^Col1a^+^ cells in renal tissue. Scale bar, 20 μm. **(G)** Western blotting detected the MKL1 protein level in MKL1^ΔFib^ and the MKL1^f/f^ renal resident fibroblasts. N = 3 mice. Data represent mean ± SD. ^*^ *p* < 0.05, two-tailed *t*-test.

In order to delete MKL1 specifically in resident fibroblasts, the *Mkl1*-flox mice were crossed to the *Col1a2*-Cre^ERT2^ mice, which upon injection of tamoxifen allow efficient removal the floxed allele in cells that express collagen type I alpha 2 chain (Col1a2) (Zheng et al., 2002) (Figure 1A). Immunofluorescence staining performed with paraffin embedded cardiac sections using an anti-MKL1 antibody and an anti-FSP1 antibody showed that there were fewer double MKL1^+^FSP1^+^ cells in the MKL1^ΔFib^ heart than in the MKL1^f/f^ heart (Figure 1B). Moreover, staining with antibodies raised against another fibroblast marker Col1a2 also identified the knockout efficiency of MKL1 in MKL1^ΔFib^ mice. As shown in Figure 1C, MKL1 expression in fibroblasts was dramatically decreased in MKL1^ΔFib^ mice, compared with MKL1^f/f^ mice. Alternatively, primary cardiac fibroblasts were isolated from the MKL1^ΔFib^ heart and the MKL1^f/f^ heart. Western blotting showed that MKL1 protein levels were significantly lower in the MKL1^ΔFib^ cells than in the MKL1^ΔFib^ cells (Figure 1D). Similar experiments were performed and verified that MKL1 was specifically deleted in renal resident fibroblasts (Figures 1E–G).

### Megakaryocytic Leukemia 1 Deletion in Resident Fibroblasts Attenuates Cardiac Fibrosis

**FIGURE 2:**
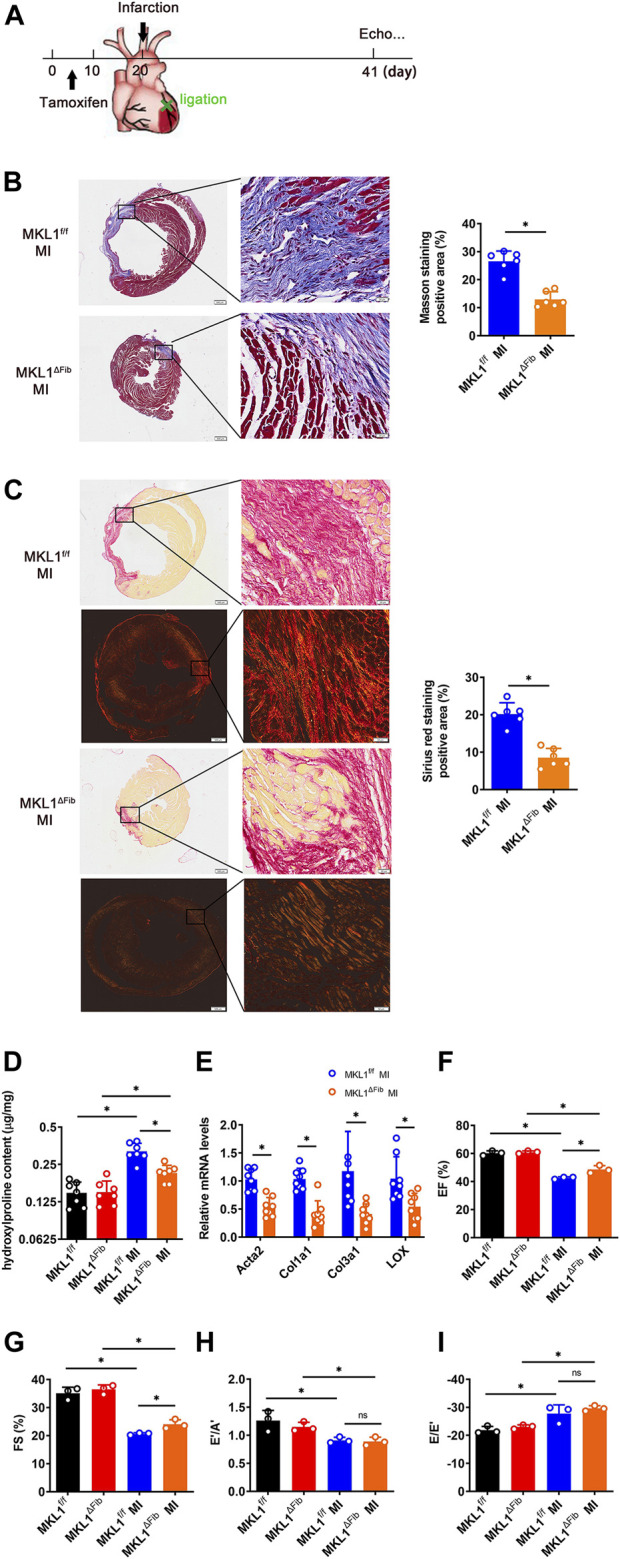
MKL1 deletion in resident fibroblasts attenuates cardiac fibrosis. **(A)** Schematic diagram of myocardial infarction (MI)-induced cardiac fibrosis in MKL1^f/f^ and MKL1^ΔFib^ mice. Tamoxifen (50 mg/kg/d) was injected into mice by intraperitoneal injection (*i.p.*) for 10 days. The mice were then subjected to MI surgery for 21 days. **(B)** Masson’s trichrome staining. Scale bar, 500 μm. N = 6 mice. **(C)** Picrosirius red staining under white light or polarized light. Scale bar, 500 μm. N = 6 mice. **(D)** Hydroxylproline level were detected by test kit. N = 7 mice. **(E)** Pro-fibrogenic genes were measured by RT-qPCR. N = 8 mice. **(F)** Ejection fraction (EF) values. N = 3 mice. **(G)** Fractional shortening (FS) values. N = 3 mice. **(H)** Late diastolic transmitral flow velocity (E’/A′) values. N = 3 mice. **(I)** Early diastolic mitral annular velocity (E/E′) values. N = 3 mice. Data represent mean ± SD. ^*^ *p* < 0.05, two-tailed *t*-test.

We evaluated the effect of MKL1 deletion in resident fibroblasts on cardiac fibrosis in a classic model in which the left anterior descending artery was permanently ligated to induce myocardial infarction (MI, Figure 2A); significant cardiac fibrosis typically develops within 7 days of the surgical procedure (Cleutjens et al., 1995). Masson’s trichrome staining (Figure 2B) and picrosirius red (Figure 2C) staining both showed significantly diminished fibrotic areas in the MKL1^ΔFib^ hearts compared to the MKL1^f/f^ hearts. In addition, collagen fiber size and alignment with picrosirius staining can be estimated under polarized light, which is correlated with collagen cross-linking and maturation (Laure Rittié, 2017). Thus, we assessed the collagen packing by visualization under polarized light. As shown in Figure 2C, the collagen fiber size, detected by polarized light, in MKL1^ΔFib^ mice was significantly decreased compared to the MKL1^f/f^ mice. Hydroxylproline quantification confirmed that the accumulation of collagenous tissues was reduced in the MKL1^ΔFib^ hearts compared to the MKL1^f/f^ hearts (Figure 2D). Quantitative PCR analysis revealed that the expression levels of several pro-fibrogenic molecules, including α-SMA (encoded by *Acta2*), collagen type I (encoded by *Col1a1*), collagen type III (encoded by *Col3a1*), and the lysyl oxidases (encoded by *Lox*) were collectively down-regulated in the heart by the loss of MKL1 in fibroblasts (Figure 2E). These data showed that cardiac fibrosis after MI in MKL1^ΔFib^ mice were significantly attenuated, compared with that in MKL1^f/f^ mice.

Next, echocardiographic measurements were taken to gauge the potential impact of MKL1 deletion in resident fibroblasts on post-MI heart functions. As shown in Figure 2F and Figure 2G, the MKL1^ΔFib^ mice had slightly better systolic functions, measured by ejection fraction (EF) and fractional shortening (FS), than the MKL1^f/f^ mice. However, no difference in diastolic functions, as measured by late diastolic transmitral flow velocity (E’/A′, Figure 2H) and the ratio of mitral peak velocity of early filling to early diastolic mitral annular velocity (E/E’, Figure 2I), was discernable between the MKL1^ΔFib^ mice and the MKL1^f/f^ mice.

### Megakaryocytic Leukemia 1 Deletion in Resident Fibroblasts Attenuates Renal Fibrosis

**FIGURE 3:**
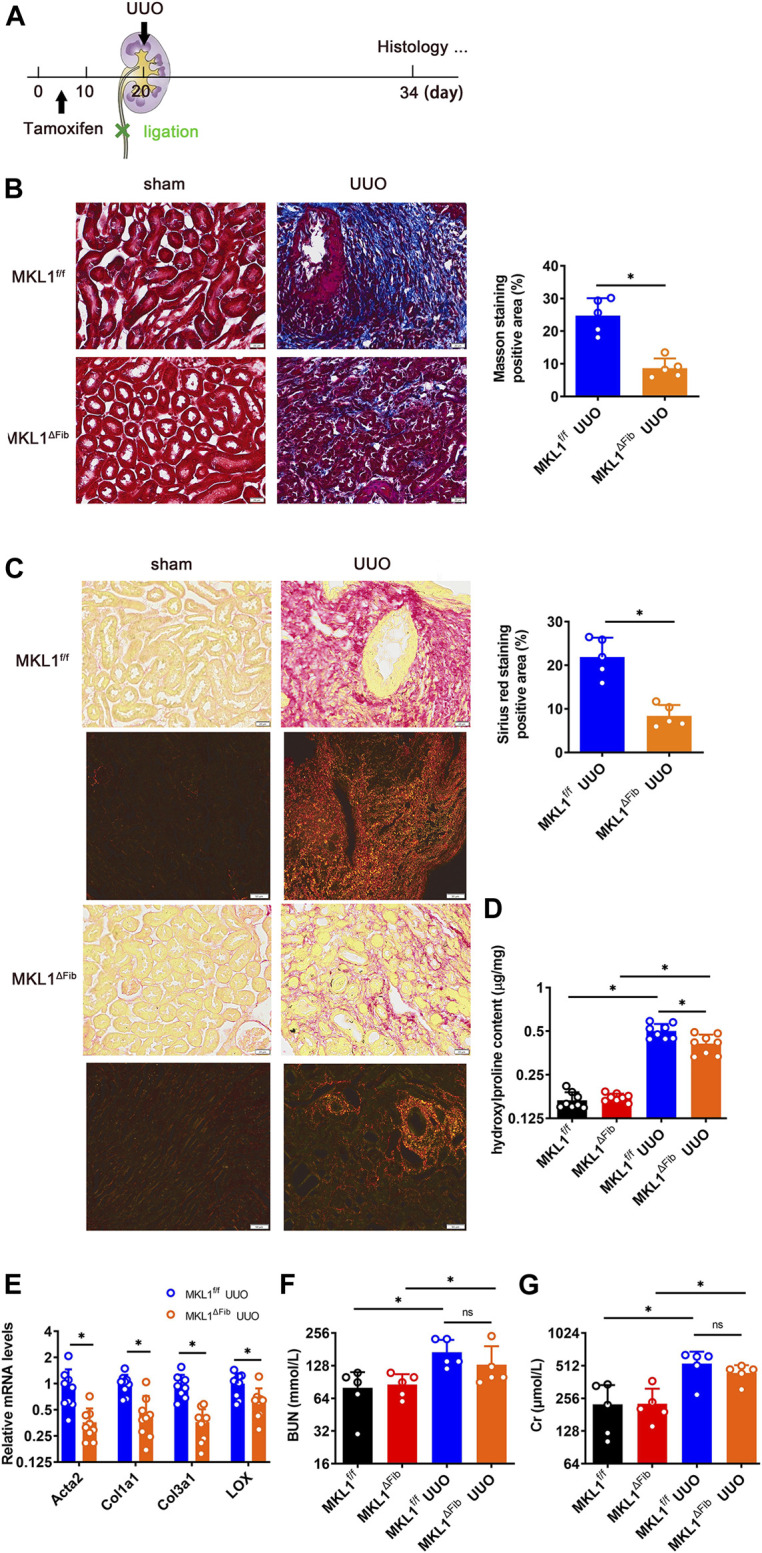
MKL1 deletion in resident fibroblasts attenuates renal fibrosis. **(A)** Schematic diagram of unilateral ureteral obstruction (UUO)-induced renal fibrosis in MKL1^f/f^ and MKL1^ΔFib^ mice. Tamoxifen (50 mg/kg/d) was injected into mice by intraperitoneal injection (*i.p.*) for 10 days. The mice were then subjected to UUO surgery for 14 days. **(B)** Masson’s trichrome staining. Scale bar, 20 μm. N = 5 mice. **(C)** Picrosirius red staining under white light or polarized light. Scale bar, 20 μm. N = 5 mice. **(D)** Hydroxylproline level were detected by test kit. N = 8 mice. **(E)** Pro-fibrogenic genes were measured by RT-qPCR. N = 9 mice. **(F)** Urine urea nitrogen (BUN) levels. N = 5 mice. **(G)** Urine creatinine (Cr) levels. N = 5 mice. Data represent mean ± SD. ^*^ *p* < 0.05, two-tailed *t*-test.

In order to evaluate the effect of MKL1 deletion in resident fibroblasts on renal fibrosis, the procedure of unilateral ureteral obstruction (UUO) was performed (Figure 3A); significant renal fibrosis can be detected within a week following the surgery (Klahr and Morrissey, 2002; Chevalier et al., 2009). Similar to the model of myocardial fibrosis, attenuation of renal fibrosis was detected in the MKL1^ΔFib^ kidneys compared to the MKL1^f/f^ kidneys, as evidenced by Masson’s trichrome staining (Figure 3B), picrosirius red staining (Figure 3C), and quantification of renal hydroxylproline levels (Figure 3D). Moreover, as shown in Figure 3C, polarized light results found the collagen fiber size in the MKL1^ΔFib^ kidneys was dramatically decreased, compared with MKL1^f/f^ kidneys. Expression levels of pro-fibrogenic genes including *Acta2*, *Col1a1*, *Col3a1* and *Lox* were lower in the MKL1^ΔFib^ kidneys than the MKL1^f/f^ kidneys as revealed by qPCR (Figure 3E), confirming the obvious trend of improvement in MKL1^ΔFib^ kidneys.

To determine the impact of MKL1 deletion in resident fibroblasts on renal functions, the plasma levels of urea nitrogen (BUN) and creatinine (Cr) were determined. As shown in Figure 3F and Figure 3G, plasmas BUN levels and Cr levels were comparable between the MKL1^ΔFib^ mice and the MKL1^f/f^ mice, suggesting that fibroblast resident MKL1 probably does not contribute to clearance of metabolic waste by the glomeruli.

### Megakaryocytic Leukemia 1 Deletion in Resident Fibroblasts Attenuates Pulmonary Fibrosis

**FIGURE 4:**
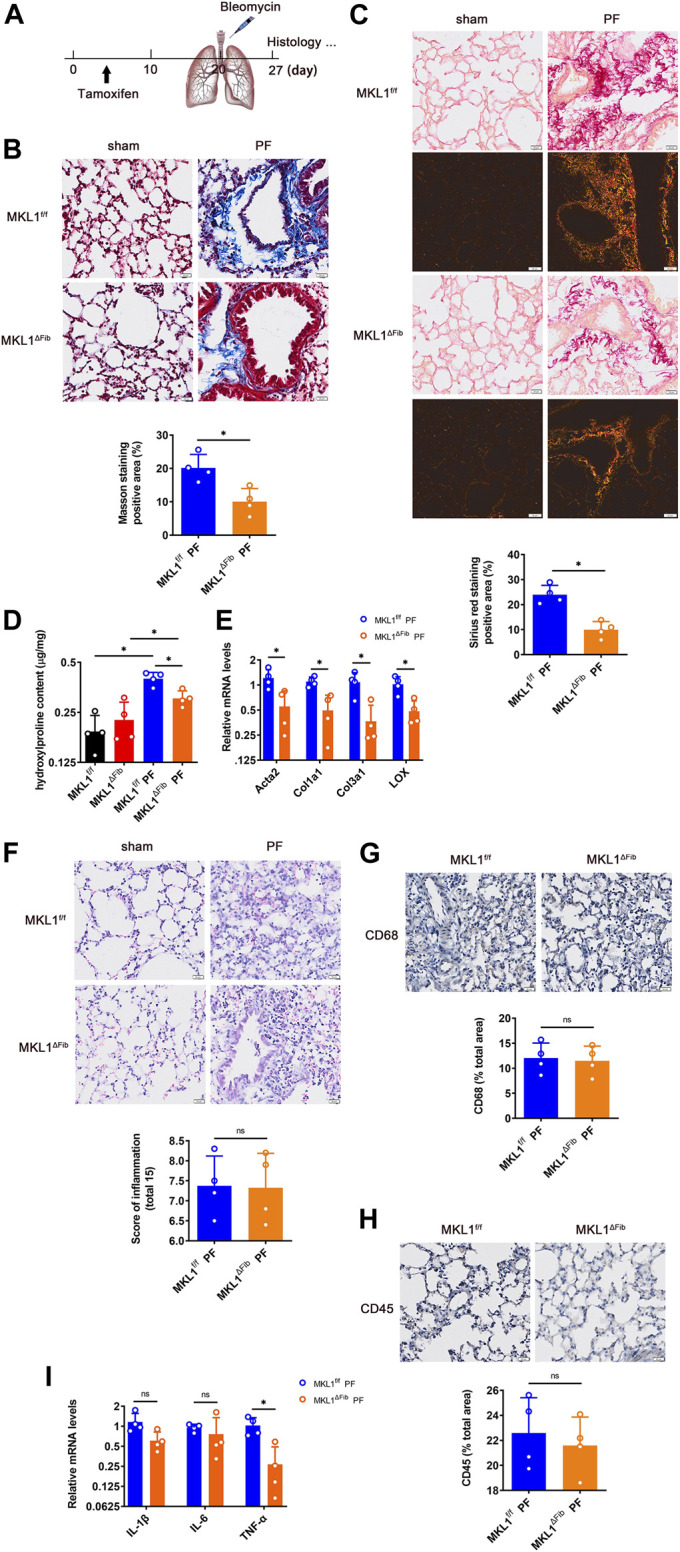
MKL1 deletion in resident fibroblasts attenuates pulmonary fibrosis. **(A)** Schematic diagram of bleomycin (5 mg/kg)-induced pulmonary fibrosis in MKL1^f/f^ and MKL1^ΔFib^ mice. Tamoxifen (50 mg/kg/d) was injected into mice by intraperitoneal injection (*i.p.*) for 10 days. The mice were then subjected with bleomycin instillation for 7 days. **(B)** Masson’s trichrome staining. Scale bar, 20 μm. **(C)** Picrosirius red staining under white light or polarized light. Scale bar, 20 μm. **(D)** Hydroxylproline levels were detected by test kit. **(E)** Pro-fibrogenic genes were measured by RT-qPCR. **(F)** H&E staining. Scale bar, 20 μm. Immunohistochemical staining detected the CD68 levels **(G)** and the CD45 levels **(H)**. Scale bar, 20 μm. **(I)** Pro-inflammatory cytokines in bronchoalveolar lavage fluid (BALF) were detected by RT-qPCR. N = 4 mice. Data represent mean ± SD. ^*^ *p* < 0.05, two-tailed *t*-test.

We next investigated the how MKL1 deletion in resident fibroblasts might alter pulmonary fibrosis. To this end, a classic model of bleomycin instillation was performed (Figure 4A); typically, an acute inflammatory response takes place in the lungs within 3 days of bleomycin administration, which is followed by interstitial fibrosis (Moeller et al., 2008). Masson’s trichrome staining (Figure 4B) and picrosirius red staining (Figure 4C) clearly indicated that interstitial fibrosis was far less extensive in the MKL1^ΔFib^ lungs than in the MKL1^f/f^ lungs. Figure 4C also showed the down-regulation of collagen fiber size in MKL1^ΔFib^ lung under polarized light. Quantification of hydroxylproline levels (Figure 4D) and qPCR analysis of pro-fibrogenic gene expression levels (Figure 4E) confirmed an amelioration of pulmonary fibrosis in the MKL1^ΔFib^ mice than in the MKL1^f/f^ mice.

On the other hand, H&E staining (Figure 4F) indicated that gross pulmonary morphology was not significantly different between the two groups. Immunohistochemical staining performed with an anti-CD68 antibody (Figure 4G), which labels macrophages, and an anti-CD45 antibody (Figure 4H), which labels granulocytes, showed similar levels of immune infiltrates in the MKL1^ΔFib^ lungs and in the MKL1^f/f^ lungs. Finally, when expression of pro-inflammatory cytokines in the cells from the bronchoalveolar lavage fluid (BALF) was examined, it was found that levels of tumor necrosis factor (*Tnfa*), but not interleukin 1 (*Il1b*) or interleukin 6 (*Il6*), were lower in the MKL1^ΔFib^ mice than in the MKL1^f/f^ mice (Figure 4I).

## Discussion

Genetic lineage tracing has enabled the delineation of cellular origins underlying a host of physiological and pathophysiological processes including tissue fibrosis (Baron and van Oudenaarden, 2019). Landmark studies, aided by the lineage tracing tools, have shown that resident fibroblasts are the major or, in some cases, the predominant source from which myofibroblasts arise to mediate the pro-fibrogenic response in various tissues (Sato and Yanagita, 2017; Gibb et al., 2020; Freeberg et al., 2021). MKL1 is a master regulator of tissue fibrosis as evidenced by the observations that global MKL1 deletion or inhibition mitigates fibrosis in the heart (Small et al., 2010; Weng et al., 2015), in the lungs (Zhou et al., 2013; Bernau et al., 2015; Sisson et al., 2015), and in the kidneys (Xu et al., 2015; Bialik et al., 2019; Mao et al., 2020b). Here we present data to show that mice harboring resident fibroblast-specific MKL1 deletion display attenuated tissue fibrosis in several different models suggesting that resident fibroblast MKL1 is sufficient to drive a pro-fibrogenic response *in vivo*.

We show here that less extensive cardiac fibrosis occurred in the MKL1^ΔFib^ mice than in the MKL1^f/f^ mice following myocardial infarction. This observation echoes the finding of a previous report by Small et al. that demonstrates an attenuation of cardiac fibrosis in the global MKL1 deficient (KO) mice (Small et al., 2010). Of note, Small et al. reported that global MKL1 deletion improved the post-MI systolic heart function but without statistical significance whereas our data suggest that fibroblast-specific MKL1 deletion significantly elevated post-MI systolic function (Figures 2F,G). It is likely MKL1 outside the fibroblast compartments may play a protective role during heart injuries such that the removal of MKL1 from this compartment may neutralize that beneficial effects conferred by deletion of MKL1 in fibroblasts. It is not clear at this point what the identity of this compartment might be. There are many factors that can affect ejection fraction and fractional shortening, such as myocardial microvascular function (Qianwen Zhao. et al., 2019), cardiomyocyte function (Schiattarella et al., 2019), and skeletal muscle mitochondrial function (Anupam A Kumar et al., 2019), the regulation of which MKL1 may not contribute to. Thus, the effect of fibroblast-specific MKL1 deletion on the recovery of ejection fraction and fractional shortening was understandably small. It has been previously shown targeted deletion of MKL1 from either endothelial cells (Weng et al., 2015) or myelocytic cells (Liu et al., 2021a; Liu et al., 2021b) or mature cardiomyocytes (Wu T. et al., 2020) could potentially mitigate the suppression of systolic heart function post injuries. On the other hand, recent studies have indicated that cytoskeletal remodeling, likely in cardiac precursor cells, is essential for heart regeneration post-injury (Morikawa et al., 2015). MKL1 is considered as a master regulator of actin reshuffling in the cells (Olson and Nordheim, 2010). It is plausible to speculate that MKL1 may play a protective role during heart injury by promoting the regenerating potential of precursor cells.

At the initial stage of ligation, one healthy kidney can indeed compensate for the function of the other, injured kidney. However, with the extension of ligation time, this compensatory effect will gradually disappear. Moreover, the glomeruli filtration as evidenced by the measurements of plasma BUN and Cr levels was altered at 2 weeks after UUO (Zhaohui Wang et al., 2020). Our data show that resident fibroblast MKL1 did not significantly alter glomeruli filtration as evidenced by the measurements of plasma BUN and Cr levels. This is because there are many complex factors that affect BUN and Cr levels. Another explanation is the animals in the present study were only observed for 2 weeks following the surgical procedure. Thus, the permanent architectural changes taking place in patients with chronic kidney disease (CKD) or end-stage renal disease (ESRD) were not faithfully recapitulated. In contrast, Xu et al. have previously shown that global MKL1 deletion significantly ameliorated glomeruli function in a model of diabetic nephropathy (Xu et al., 2015). A wealth of data suggests that there is generally an inverse relationship between renal fibrosis and glomerular filtration functions (Djudjaj and Boor, 2019). Therefore, one would expect to record an improvement of glomerular function as a result of reduced fibrosis in the MKL1^ΔFib^ mice. One explanation why this was not the case could be that the animals in the present study were only observed for 2 weeks following the surgical procedure. Thus, the permanent architectural changes taking place in patients with chronic kidney disease (CKD) or end-stage renal disease (ESRD) were not faithfully recapitulated, which indicates that the UUO model is an imperfect model to study renal fibrosis for it does not encompass the full pathology in humans (Becker and Hewitson, 2013). In a similar vein, it is noteworthy that we did not observe any difference between the MKL1^ΔFib^ mice and in the MKL1^f/f^ mice in terms of diastolic heart functions (Figures 2H,I) despite the fact that excessive fibrosis tends to increase myocardial rigidity and interferes with the diastolic functions (Burlew and Weber, 2002). Future studies should aim to evaluate the long-term effects of MKL1 deletion in fibroblasts on organ functions.

We have similarly found that fibroblast-specific MKL1 deletion attenuated pulmonary fibrosis but minimally altered the inflammatory response in the lungs. By comparison, an earlier study has found that global MKL1 deletion protects the mice from lipopolysaccharide (LPS) induced pulmonary inflammation in a model of septicemia owing to the regulatory role of MKL1 in macrophages (Yu et al., 2017). However, we did observe a difference in TNF-α expression levels in the cells collected from BALF (Figure 4I). BALF cells mostly are of the immune lineages including macrophages, lymphocytes, and granulocytes (Sterclova et al., 2019). Therefore, the decrease in TNF-α expression could be simply construed as a reduction in immune cell trafficking in the MKL1^ΔFib^ lungs, which raises an intriguing point that MKL1 in resident fibroblasts is able to regulate not only pro-fibrogenic genes (e.g., collagen) but chemoattractive genes to promote immune cell infiltration. Indeed, pulmonary fibroblasts can function as a source from which chemokines are derived (Boyd et al., 2020). Coincidently, MKL1 has been shown to directly activate the transcription of a slew of chemokines (Yu et al., 2014; Song et al., 2017). Although immunohistochemical staining data indicate that infiltration of neither CD68^+^ macrophages nor CD45^+^ granulocytes in the lungs was significantly altered by MKL1 deletion in fibroblasts (Figures 4G,H), the possibility that specific sets of immune cell populations could be modulated by MKL1-dependent and fibroblast-derived chemokines cannot be completely excluded and deserves further investigation.

In summary, we provide evidence to implicate resident fibroblast derived MKL1 as a sufficient driving force in tissue fibrosis. Despite the clarification that the pro-fibrogenic ability of MKL1 likely emanates from fibroblasts, several outstanding questions remain to be answered. First, the effects of fibroblast-specific MKL1 deletion on tissue fibrosis have been thoroughly investigated through long-term observations because only acute models were employed in the present study. In addition, the conclusion has not been validated in multiple models of tissue fibrosis that typically represent the far complex etiologies in humans. Second, there is a great deal of heterogeneity in fibroblast populations, which is partly determined and shaped by tissue origins (Lynch and Watt, 2018). Although the MKL1^f/f^ mice seem to exhibit similar phenotypes in different models of tissue fibrosis, it is unlikely that the transcriptomes of cardiac fibroblasts, pulmonary fibroblasts, and renal fibroblasts might be uniformly altered by the loss of MKL1. Recent advances in single-cell RNA-sequencing have greatly facilitated the fine-dissecting the dynamic transcriptomic changes in a wide ranges of tissues and organs (Fu et al., 2021; Liang et al., 2021; Ma et al., 2021; Xie et al., 2021; Zhao et al., 2021). Therefore, a comparison of gene expression profiles of the MKL1-null fibroblasts using this state-of-the-art technique in different organs could shed additional insight on the mode of action for MKL1. These caveats will need to be addressed in future studies so that the field can inch closer to understanding the holistic picture wherein MKL1 contributes to tissue fibrosis.

## Data Availability

The original contributions presented in the study are included in the article/supplementary Material, further inquiries can be directed to the corresponding authors.
